# Relevance of CD6-Mediated Interactions in the Regulation of Peripheral T-Cell Responses and Tolerance

**DOI:** 10.3389/fimmu.2017.00594

**Published:** 2017-05-30

**Authors:** Marta Consuegra-Fernández, Mario Martínez-Florensa, Fernando Aranda, José de Salort, Noelia Armiger-Borràs, Teresa Lozano, Noelia Casares, Juan José Lasarte, Pablo Engel, Francisco Lozano

**Affiliations:** ^1^Immunoreceptors of the Innate and Adaptive System Group, Institut d’Investigacions Biomèdiques August Pi i Sunyer (IDIBAPS), Barcelona, Spain; ^2^Immunology Unit, Department of Biomedical Sciences, School of Medicine, University of Barcelona, Barcelona, Spain; ^3^Program of Immunology and Immunotherapy, Center for Applied Medical Research (CIMA), University of Navarra, Pamplona, Spain; ^4^Immunology Department, Centre de Diagnòstic Biomèdic, Hospital Clínic of Barcelona, Barcelona, Spain

**Keywords:** CD6 deficiency, T_reg_ cells, chronic graft-versus*-*host disease, mixed lymphocyte reaction, lupus-like mouse model, autoimmunity, peripheral tolerance

## Abstract

The CD6 lymphocyte receptor has been involved in the pathophysiology of different autoimmune disorders and is now considered a feasible target for their treatment. *In vitro* data show the relevance of CD6 in the stabilization of adhesive contacts between T-cell and antigen-presenting cells, and the modulation of T-cell receptor signals. However, the *in vivo* consequences of such a function are yet undisclosed due to the lack of suitable genetically modified animal models. Here, the *in vitro* and *in vivo* challenge of CD6-deficient (CD6^−/−^) cells with allogeneic cells was used as an approach to explore the role of CD6 in immune responses under relative physiological stimulatory conditions. Mixed lymphocyte reaction (MLR) assays showed lower proliferative responses of splenocytes from CD6^−/−^ mice together with higher induction of regulatory T cells (T_reg_, CD4^+^CD25^+^FoxP3^+^) with low suppressive activity on T and B-cell proliferation. In line with these results, CD6^−/−^ mice undergoing a lupus-like disorder induced by chronic graft-versus-host disease (cGvHD) showed higher serum titers of anti-double-stranded DNA and nucleosome autoantibodies. This occurred together with reduced splenomegaly, which was associated with lower *in vivo* bromodesoxyuridine incorporation of spleen cells and with increased percentages of spleen follicular B cells (B2, CD21^+^CD23^hi^) and T_reg_ cells. Interestingly, functional analysis of *in vivo-*generated CD6^−/−^ T_reg_ cells exhibited defective suppressive activity. In conclusion, the data from MLR and cGvHD-induced lupus-like models in CD6^−/−^ mice illustrate the relevance of CD6 in T (and B) cell proliferative responses and, even more importantly, T_reg_ induction and suppressive function in the *in vivo* maintenance of peripheral tolerance.

## Introduction

CD6 was one of the first lymphocyte surface markers identified following the advent of monoclonal antibody (mAb) technology (1). The fact that CD6 was preferentially expressed by all T cells, as well as by a small subset of mature B cells (B1a cells) involved in the production of low affinity polyreactive autoantibodies, soon led to early though discontinued attempts to treat certain autoimmune disorders (e.g., rheumatoid arthritis, psoriasis, and multiple sclerosis) using mouse anti-CD6 mAbs (2). The recent marketing of a humanized anti-CD6 mAb (itolizumab) (3, 4), together with the identification of *CD6* gene as a multiple sclerosis susceptibility locus (5, 6), has renewed the interest in the study of this relatively neglected lymphocyte receptor. Due to the unavailability of genetically modified animal models targeting *cd6* gene, the rationale for CD6-based therapeutic strategies mostly stems from *in vitro* data. However, when translated into more complex *in vivo* systems, *in vitro* results have sometimes been misleading. This is a key lesson learned from the sister molecule CD5, since full characterization of its biological role and development of its therapeutic potential could not be realized until CD5-knockout mouse models became available (7).

CD6 is a 105–130 kDa transmembrane glycoprotein expressed by all mature and developing T lymphocytes, a subgroup of natural killer and B (B1a) cells (1, 8, 9), some hematopoietic cell precursors (10) and certain brain cells (11). The main CD6 ligand is CD166/ALCAM (activated leukocyte cell adhesion molecule), a broadly expressed cell adhesion molecule of the immunoglobulin superfamily present on thymic epithelial cells, endothelial cells, and antigen-presenting cells (APC) such as dendritic cells, macrophages, and B cells (12). The CD6–CD166/ALCAM interaction has recently been structurally solved (13), and it is long known to be critical for the stabilization and maturation of the immunological synapse (IS) (14–16), as well as for transmigration of T cells to the central nervous system in autoimmune encephalomyelitis (17) and arthritis (18) lesions. Previous reports also point to a relevant role for CD6 in T-cell development (19) and in the regulation of peripheral T-cell activation (14–16, 20, 21).

CD6 has a cytoplasmic tail devoid of intrinsic catalytic activity, but includes consensus motifs for Tyr (9) and Thr/Ser (22, 23) phosporylation and interaction with different intracellular signaling effectors such as mitogen-activated protein kinases (24), SH2 domain-containing leukocyte protein of 76 kDa (SLP-76) (21, 25) and syntenin (26). This allows CD6 modulating the activation responses triggered through the T-cell receptor (TCR)/CD3 complex to which it is physically associated at the center of the IS (14, 15). Whether CD6-dependent signaling events modulate positively or negatively T-cell activation in a manner similar to that reported to the closely related CD5 lymphocyte receptor is a debatable matter (7). Most anti-CD6 mAbs exert co-mitogenic effects on T cells, suggesting that CD6 may transduce costimulatory signals (7). However, such signals may induce opposing effects (either activating or inhibitory) depending on the experimental system used. This is the case with the UMCD6 mAb, which is co-mitogenic in autologous mixed lymphocyte reaction (MLR) (27), but inhibits the proliferation of antigen-specific and auto-reactive cloned T cells (28). Moreover, attenuation of TCR/CD3-mediated early and late T-cell activation responses by CD6 overexpression has been reported (20), suggesting that it might play a negative modulatory role.

Recent available information from a CD6-deficient (CD6^−/−^) mouse model shows the *in vivo* relevance of CD6 in (i) T-cell development by increasing the threshold for thymocyte negative selection and (ii) the homeostasis of some antigen-experienced peripheral T-cell subsets such as effector/memory T cells (T_EM_) and regulatory T cells (T_reg_), the latter being also dysfunctional (29). However, most *in vitro* studies were conducted under supraphysiological TCR-stimulation conditions—by direct mAb-induced cross-linking of the TCR/CD3 complex—and did not take into consideration the role assigned to the CD6–CD166/ALCAM interaction during adhesive cell-to-cell contacts necessary for proper T-cell activation. To further improve our understanding of the biological role played by CD6 in the regulation of peripheral immune responses, we investigated the *in vivo* and *in vitro* consequences of CD6 deficiency during allogeneic stimulation—a well-known model of cell contact-dependent antigenic challenge. To this end, coisogenic major histocompatibility complex (MHC) class II (Ia)-incompatible allogeneic B6.C-H-2^bm12^/KhEg (bm12) splenocytes were used for MLR assays, as well as for induction of a lupus-like disorder due to chronic graft-versus-host disease (cGvHD) (30). The results presented herein further underscore the significant role played by CD6 signaling and/or CD6–CD166/ALCAM adhesive interactions not only in the induction but also the proper regulation of peripheral immune responses.

## Materials and Methods

### Mice

B6.C-*H2*^bm12^/KhEg (bm12) mice were obtained from The Jackson Laboratory (id. 003625). CD6-deficient (CD6^−/−^) mice were generated in C57BL/6N background as reported elsewhere (29). Mice were housed under specific pathogen-free conditions. Eight- to twelve-week-old CD6^−/−^ and wild-type (CD6^+/+^) C57BL/6N females were used in all experiments following approved protocols by the University of Barcelona Animal Experimentation Ethical Committee.

### Induction of cGvHD

Following a previously described protocol (30), cGvHD was induced in sex and age-matched recipient CD6^−/−^ and CD6^+/+^ mice by intraperitoneal (i.p.) injection of pooled 10^8^ bm12 splenocyte suspensions, previously disaggregated by filtering through a cell strainer. Mice were weighted and bled by facial vein weekly. Sera samples were stored at −20°C until use.

### Serum Autoantibody Measurements

Serum levels of anti-dsDNA and anti-nucleosoma antibodies were measured by ELISA as previously reported (31). Briefly, 96-well plates (Nunc, Thermo Fisher Scientific, Denmark) were coated with nucleosome (3 µg/mL, Arotec) or dsDNA (10 µg/mL, Alpha Diagnostic International) in carbonate buffer pH 9.6, and incubated overnight (o/n) at 4°C. After washing thrice with phosphate-buffered saline plus 0.5% Tween-20 (PBS-T), plates were blocked with 5% bovine seroalbumin in PBS for 1 h at room temperature (RT) and further incubated for 2 h at RT with mouse sera (1/100 dilution) or mouse antibody anti-dsDNA (HPS22; ImmunoTools) used as standard. Plates were then washed thrice with PBS-T and, after 1 h incubation at RT with horseradish peroxidase-conjugated anti-mouse IgG (1/2,000, Jackson ImmunoResearch) further developed with 3,3′,5,5′-tetramethylbenzidine substrate solution (BD Biosciences). The colorimetric reaction was stopped with 0.5 M H_2_SO_4_ (50 µL) and read at 450 and 620 nm λ on a spectrophotometer (Epoch, Biotek).

### Cytokine Assays

Mouse cytokine (IL-2, IFN-γ) levels in cell culture supernatants were measured by ELISA (BD OptEIA sets, BD Biosciences) following manufacturer’s instructions.

### Flow Cytometry Analyses

Surface staining of cells was performed by suspending them in blocking solution (10% fetal calf serum in PBS) for 30 min at 4°C in the presence of the following fluorescent-labeled mAbs: anti-B220 (RA3-6B2, eBioscience), anti-CD3 (145-2C11, TONBO Bioscience), anti-CD4 (GK1.5, BioLegend), anti-CD8 (53-6.7, TONBO Bioscience), anti-CD25 (PC61.5, eBioscience), anti-CD21 (7G6, eBioscience), anti-CD23 (B3B4, eBioscience) and anti-LAP (TW7-16B4, BioLegend), anti-CXCR5 (SPRCL5, eBioscience), anti-PD1 (J43, eBioscience), anti-CD138 (281-2, BioLegend), anti-CD19 (1D3, TONBO Bioscience), anti-GL-7 (GL7, eBioscience), anti-IgM (eb121-15-F9, eBioscience), anti-IgD (11-26c, eBioscience), anti-CD40L (MR1, eBioscience), and anti-CD69 (H1.2F3, BioLegend). For intracellular cytokine staining, cells were exposed to monensin, fixed and permeabilized using the BD Cytofix/Cytoperm Plus Kit with BD GolgiStop™ (BD Biosciences) following manufacturer’s indications, and then stained with specific fluorescent-labeled mAbs: anti-IFN-γ (FITC-XMG1.2, TONBO Bioscience) and anti-IL-2 (PE-JES6-5H4, BD Pharmingen). For FoxP3 staining, the Anti-Mouse/Rat Foxp3 PE Staining Set (eBioscience) was used following manufacturer’s indications. Flow cytometry analyses were performed on a FACS Canto II equipped with CellQuest (BD Biosciences) and Flowjo 8.7 software.

### MLR Assays

γ-irradiated (1 cycle, 1,000 rads) stimulator bm12 splenocytes (2 × 10^5^ cells/well) were cocultured in U-bottomed 96-well plates (Biofil) for 5 days with responder (non-irradiated) total splenocytes or CD4^+^ T cells (10^5^ cells/well) from CD6^−/−^ or CD6^+/+^ mice. In some cases, Pokeweed mitogen (PWM, Sigma) or anti-CD28 mAb (TONBO biosciences) were added as costimulatory agents. Total splenocyte suspensions were obtained by disaggregation through cell strainer and CD4^+^ T cells were further isolated with Dynabeads Untouched Mouse CD4 T cells kit (Invitrogen) following manufacturer’s instructions. As a negative control, CD6^−/−^ and CD6^+/+^ splenocytes were irradiated and used as stimulators of total splenocytes or isolated CD4^+^ T cells from CD6^−/−^ and CD6^+/+^, respectively. Cells were suspended in RPMI 1640 with l-glutamine (Lonza), 10% fetal calf serum (BioWest), 100 U/mL penicillin, 100 µg/mL streptomycin, and 50 µM 2-β Mercaptoethanol (Merck), and cultured at 37°C in a humidified atmosphere with 5% CO_2_. Measurement of CD4^+^ T-cell proliferation was performed by adding 0.5 μCi/well [^3^H]-thymidine (PerkinElmer) 16 h before the end of the stimulation. Cells were then harvested in a Wallac cell harvester (Perkin Elmer) and incorporated radioactivity was measured in a Wallac Scintillation counter (Perkin Elmer) and represented as counts per minute (cpm). Flow cytometry assessment of splenocyte proliferation was carried out by using the CellTrace™ CFSE cell proliferation kit (Molecular Probes, Life Technologies) following manufacturer’s instructions.

### *In Vivo* Bromodesoxyuridine (BrdU)-Incorporation Assay

For *in vivo* detection of proliferating cells, mice undergoing cGvHD were given 1 mg BrdU (BD Pharmingen) i.p. 15 h prior to sacrifice at the end of fifth week. Spleen cells were then surface stained with different fluorescent-labeled T- and B-cell-specific mAbs, and further BrdU-incorporation assessment was done by flow cytometry using the FITC BrdU flow kit (BD Pharmingen) following manufacturer’s instructions.

### Regulatory and Conventional T-Cell Isolation and *In Vitro* Treg Suppression Assays

CD4^+^CD25^+^ T regulatory cells (T_reg_) and CD4^+^CD25^−^ T conventional cells (T_conv_) from spleen and lymph node of CD6^−/−^ and CD6^+/+^ mice were sorted by flow cytometry (FACS ARIA III, BD Biosciences) following labeling with anti-CD4 (FITC-RM4-5, TONBO Bioscience) and anti-CD25 mAb (APC-PC61.5, eBioscience). The isolated fractions were tested for purity (> 95%) and used immediately. The functionality of CD4^+^CD25^+^ T cells (T_reg_) was evaluated by measuring their ability to suppress T_conv_ (CD4^+^CD25^−^) and B-cell proliferation induced by exposure to allogeneic cells and surface IgM cross-linking, respectively. To this end, γ-irradiated stimulator bm12 splenocytes (2 × 10^5^ cells/well) were cocultured for 5 days with CD6^+/+^ T_conv_ cells (10^5^ cells/well) in the presence or absence of different ratios of T_reg_ (2 × 10^4^ or 10^4^ cells/well) from CD6^−/−^ and CD6^+/+^ mice. Cell culture conditions and cell proliferation assessment by [^3^H]-thymidine cell incorporation was performed as above. Alternatively, CFSE-labeled CD6^+/+^ B sorted cells (see *In Vitro* B and T-Cell Proliferation Assays) were stimulated for 3 days with goat F(ab’)_2_ anti-mouse IgM (10 µg/mL, Jackson ImmunoResearch) in the presence or absence of different ratios of T_reg_ (2 × 10^4^ or 10^4^ cells/well) from CD6^−/−^ and CD6^+/+^ mice, as previously mentioned. At the end of the culture period, cells were analyzed by cytometry for assessment of CFSE intensity.

### *In Vitro* Treg Induction Assay

Sorted T_conv_ cells (10^5^ cells/well) from CD6^−/−^ and CD6^+/+^ spleens were cultured for 3 days in U-bottomed 96-well plates coated with anti-CD3 mAb (145-2C11; 2 µg/mL, TONBO Bioscience) plus soluble anti-CD28 mAb (37.51; 5 µg/mL, TONBO Bioscience) in the presence of TGF-β (2 ng/mL, ImmunoTools) and IL-2 (20 ng/mL, ImmunoTools). Cells were then stained with anti-CD4 (FITC-RM4-5), anti-CD25 (APC-PC61.5), and anti-FoxP3 (PE-3G3, eBioscience) for further flow cytometry analysis of Treg (CD4^+^CD25^+^FoxP3^+^) cell induction.

### *In Vitro* B and T-Cell Proliferation Assays

Spleen B cells from CD6^−/−^ and CD6^+/+^ were FACS sorted by negative selection with anti-CD3 (APC-145-2C11, TONBO Bioscience), anti-CD11b (PE-M1770, eBioscience), and anti-CD11c (PerCPCy5.5-N418, TONBO Bioscience) mAbs. Spleen Tconv (CD4^+^CD25^−^) cells were sorted as above described. The isolated fractions were tested for purity (>95%) and CSFE-labeled as above. Purified B cells (10^5^ cells/well) were then cultured for 3 days in the presence of goat F(ab’)_2_ anti-mouse IgM fragment (10 µg/mL, Jackson ImmunoResearch) or lipopolysaccharide (LPS, 20 µg/mL, Sigma) with or without IL-4 (10 ng/mL, ImmunoTools) or anti-CD40 mAb (1C10, Biolegend; 5 µg/mL). CSFE-labeled purified CD4^+^ T cells were also cultured for 3 days in U-bottomed 96-well plates coated with anti-CD3 mAb (145-2C11; 2 µg/mL). At the end of the culture period, cells were analyzed by cytometry for assessment of CFSE intensity.

### RNA Extraction and Quantitative Real-time RT-PCR

Total RNA extraction from cell cultures was performed as previously described (32, 33). Mouse IL-2, IL-17, CD40L, IFN-γ, and β-actin expression was measured by quantitative real-time PCR using SYBR Green Real-Time PCR Master Mix kit (ThermoFisher Scientific) and the following specific primers: IL-2: Fw 5′-TGT TGA TGG ACC TAC AGG AG, Rv 5′-GTG TTG TCA GCC CTT TAG; IL-17: Fw 5′-CTG TGT CTC TGA TGC TGT TG, Rv 5′-TAT CAG GGT CTT CAT TGC GG; CD40L: Fw 5′-ACT GTG AGG AGA TGA GAA GC, Rv 5′-ACT GTA GAA CGG ATG CTG; IFN-γ: Fw 5′-TCA AGT GGC ATA GAT GTG GAA G, Rv 5′-TGG CTC TGC GGA TTT TCA TG; and β-actin: Fw 5′-CGC GTC CAC CCG CCA C, Rv 5′-CCT GCT GCC TAG GCG. The level of expression of β-actin was used to normalize gene expression.

### Statistical Analyses

Data were expressed as mean ± SD. Statistical analyses were performed by parametric *t*-tests for paired data using GraphPad Prism (GraphPad Software). *p values ≤ 0.05, **p values ≤ 0.01, and ***p values ≤ 0.001 were considered statistically significant.

## Results

### CD6^−/−^ T Cells Exhibit Low *In Vitro* Proliferative Responses to Allogeneic Stimulation

**Figure 1:**
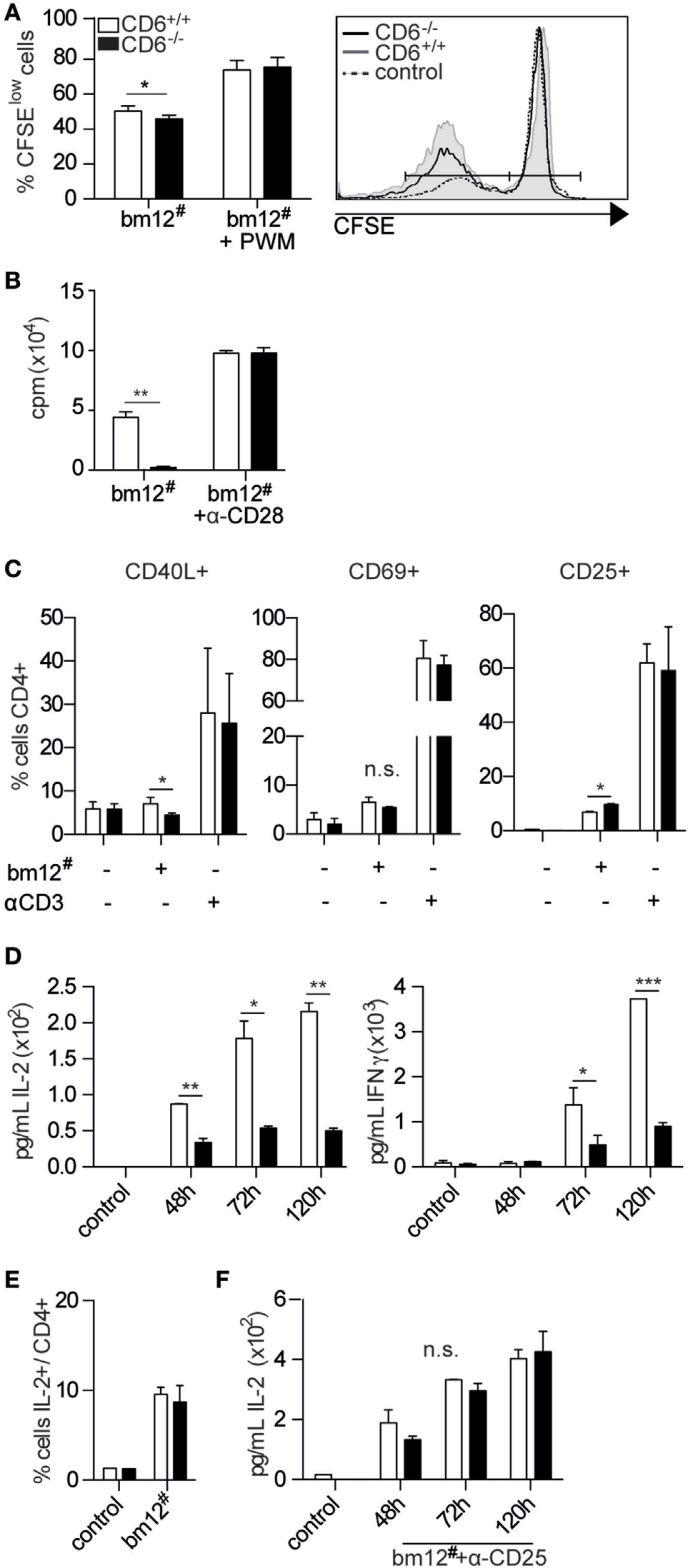
***In vitro* activation and proliferative responses of spleen CD6^−/−^ cells to allogeneic mixed lymphocyte reaction (MLR) stimulation**. **(A)** Responder CFSE-stained total splenocytes from CD6^−/−^ (solid bars) or CD6^+/+^ (empty bars) mice were cocultured for 5 days at a 2:1 ratio with irradiated total bm12 splenocytes (bm12^#^) as stimulators. Percentage (mean ± SD) of proliferating B cells (CFSE^low^) following MLR-induction alone or in the presence of Pokeweed mitogen (PWM) is shown. Data shown are triplicate samples from one representative experiment of three performed. **(B)** Spleen CD4^+^ T cells isolated by negative selection from CD6^−/−^ (solid bars) or CD6^+/+^ (empty bars) mice were cocultured for 5 days with irradiated bm12 splenocytes (bm12^#^) in the presence or absence of anti-CD28 monoclonal antibody (mAb) (α-CD28, 1 µg/mL). The bar chart shows the [^3^H]-thymidine incorporation (in cpm) of triplicate samples from one representative experiment of four performed. **(C)** Same sorted CD4^+^ T cells from **(B)** were stimulated for 24 h and analyzed for CD40L, CD69, and CD25 surface expression in CD4^+^ gated T cells by flow cytometry. A representative experiment of three performed is shown. **(D)** Bart charts representing the IL-2 (left-hand side) and IFN-γ (right-hand side) levels (pg/mL) over time from supernatants of same cocultures as in **(B)**. **(E)** Percentage (mean ± SD) of intracellular IL-2-positive cells among CD4^+^ spleen T cells from day 5 of same cocultures as in **(B)**. **(F)** IL-2 levels (pg/mL) over time from supernatants of same cocultures as in **(B)** in the presence of exogenously added blocking anti-CD25 mAb (30 µg/mL). n.s., not significant; *p < 0.05; **p < 0.01; ***p < 0.001 (Student’s *t*-test).

Available *in vitro* evidence indicates that adhesive CD6 signaling and/or adhesive CD6–CD166/ALCAM interaction is required for optimal T-cell activation and proliferation (14, 16). To further investigate *in vitro* proliferative responses of peripheral T cells from CD6^−/−^ mice under cell-to-cell contact-dependent antigenic stimulation, MLR assays were performed. To this end, irradiated bm12 (bm12^#^) splenocytes—which differ from those of C57BL/6N by three amino acids in the β-chain of their I-A molecules—were cocultured for 5 days at 2:1 ratio with non-irradiated CFSE-labeled splenocytes from CD6^−/−^ or CD6^+/+^ mice, respectively. As illustrated in Figure 1A, responder CD6^−/−^ splenocytes showed statistically significant lower proliferative responses than CD6^+/+^ ones, a fact that did not happen in the presence of costimulation with PWM—a monocyte-dependent T (and B) cell activator (34). Given that the MLR system here used is driven by MHC class II allorecognition, purified CD4^+^ spleen T cells from CD6^−/−^ and CD6^+/+^ mice were used as responders to irradiated bm12 stimulator cells at 2:1 ratio. Similar to results obtained with CFSE-labeled total splenocytes, CD4^+^ T cells from CD6^−/−^ mice showed statistically significant lower [^3^H]-thymidine incorporation than those from CD6^+/+^ (Figure 1B). Worthy of note, these proliferative differences also disappeared when an anti-CD28 mAb was added as a costimulatory agent. Moreover, no significant differences regarding apoptotic cell numbers were detected between CD6^−/−^ and CD6^+/+^ responder cells in MLR assays (data not shown).

The fact that CD6-mediated interactions are relevant to IS formation and maturation (14–16) would suggest that deficient TCR signal strength could account for the lower T-cell proliferative response to allogeneic stimulation observed in CD6^−/−^ mice. Consequently, the induction of early surface markers of T-cell activation events at mRNA and protein levels was analyzed at 7 and 24 h, respectively, following allogeneic stimulation of CD4^+^ T cells from CD6^+/+^ and CD6^−/−^ with irradiated allogeneic (bm12^#^) splenocytes. As shown in Figure 1C, CD40L and CD69 surface expression was also lower in CD6^−/−^ T cells compared to CD6^+/+^ ones, reaching statistically significant values only for the former (Figure 1D). On the contrary, CD25 surface expression was found significantly increased in CD6^−/−^ T cells. Interestingly, no significant differences were observed for any of the early activation events analyzed when CD6^−/−^ and CD6^+/+^ T cells were challenged via CD3 cross-linking, used as a positive stimulation control. Taken together, the small differences observed in early T-cell activation markers stemming from our allogeneic model do not allow unequivocally supporting attenuated signal strength as a likely underlying cause of lower T-cell proliferation observed in CD6^−/−^ mice post-MLR stimulation.

Monitoring of MLR culture supernatants from CD6^−/−^-deficient CD4^+^ T cells showed significantly lower IL-2 and IFN-γ cytokine levels at all time points analyzed when compared with their CD6^+/+^ counterparts (Figure 1D). Intracellular cytokine staining of MLR cocultures revealed that CD4^+^ T cells from CD6^−/−^ spleens did not present lower, but similar percentage of IL-2-positive cells compared to those from CD6^+/+^ mice (Figure 1E). Since these data favored the idea of increased cytokine consumption rather than defective cytokine production as responsible for low cytokine levels in MLR culture supernatants, same assays were performed in the presence of a blocking anti-CD25 (IL-2 receptor α chain, IL-2Rα) mAb. As shown in Figure 1F, addition of the anti-CD25 mAb to the MLR culture abrogated the abovementioned differences in IL-2 supernatant levels between CD6^−/−^ and CD6^+/+^ alloreactive CD4^+^ T cells, thus pointing to an accelerated cytokine consumption.

### Preserved Proliferative Responses of CD6^−/−^ T Cells to Direct Antigen-Specific Receptor Cross-linking

**Figure 2:**
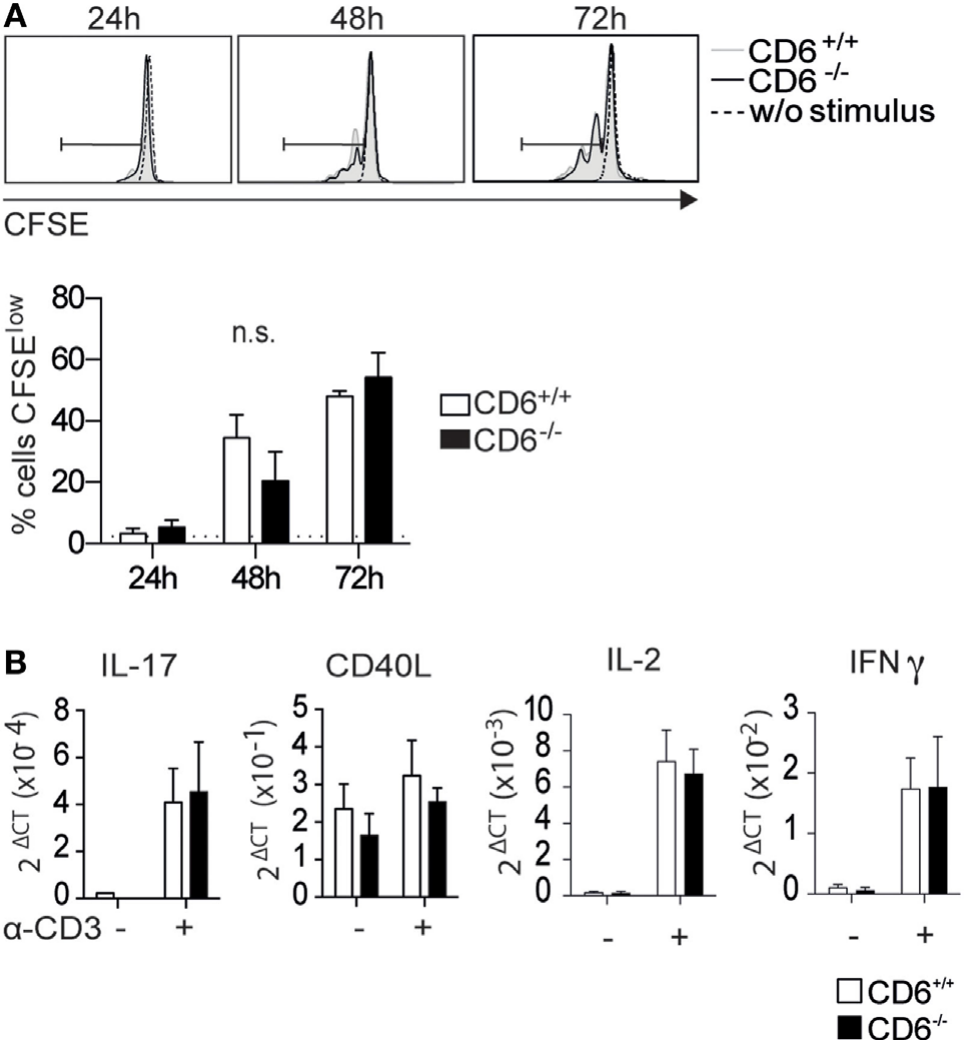
***In vitro* activation and proliferative responses of spleen CD4^+^ T cells from CD6^−/−^ and CD6^+/+^ mice to CD3 cross-linking**. **(A)** Sorted spleen CD4^+^ T cells from untreated CD6^−/−^ and CD6^+/+^ mice were CFSE-labeled and cultured for 3 days either alone or plastic-bound anti-CD3 monoclonal antibody (1 µg/mL) for further flow cytometry analysis. The histogram shows the CFSE fluorescence intensity from a representative experiment of four performed, at different time points. **(B)** CD4^+^ T cells stimulated as in **(A)** for 7 h were analyzed for mRNA expression levels of the indicated genes by real-time PCR. The bar charts represent quintuplicate mRNA values from six pooled mice, where Δ*C*_t_ indicates the difference in the threshold cycle between GADPH and target genes. n.s., not significant (Student’s *t*-test).

In light of results depicted in Figures 1C,D, it was further assessed whether cell-intrinsic defects could account for the lower *in vitro* proliferation found in CD6^−/−^ mice following allogeneic stimulation. To this end, sorted CD4^+^ T cells were CFSE-labeled and their proliferative response measured by flow cytometry upon 3-day stimulation via mAb-induced CD3 cross-linking. As shown in Figure 2A, no statistically significant differences were observed between CD6^−/−^ and CD6^+/+^ CD4^+^ T cells regarding their CD3-mediated proliferative responses. Moreover, no significant differences were also observed when mRNA expression levels for IL-2 and IFN-γ (Figure 1C), as well as IL-17, CD40L, and IL-10 (Figure 2B) were assessed from the same *in vitro* CD3-stimulated CD4^+^ T cells. Taken together, these results argue against significant T-cell-intrinsic defects associated with CD6 deficiency.

### CD6^−/−^ CD4^+^ T Cells Differentiate More Efficiently into FoxP3^+^ Cells Following Allogeneic Stimulation

**Figure 3:**
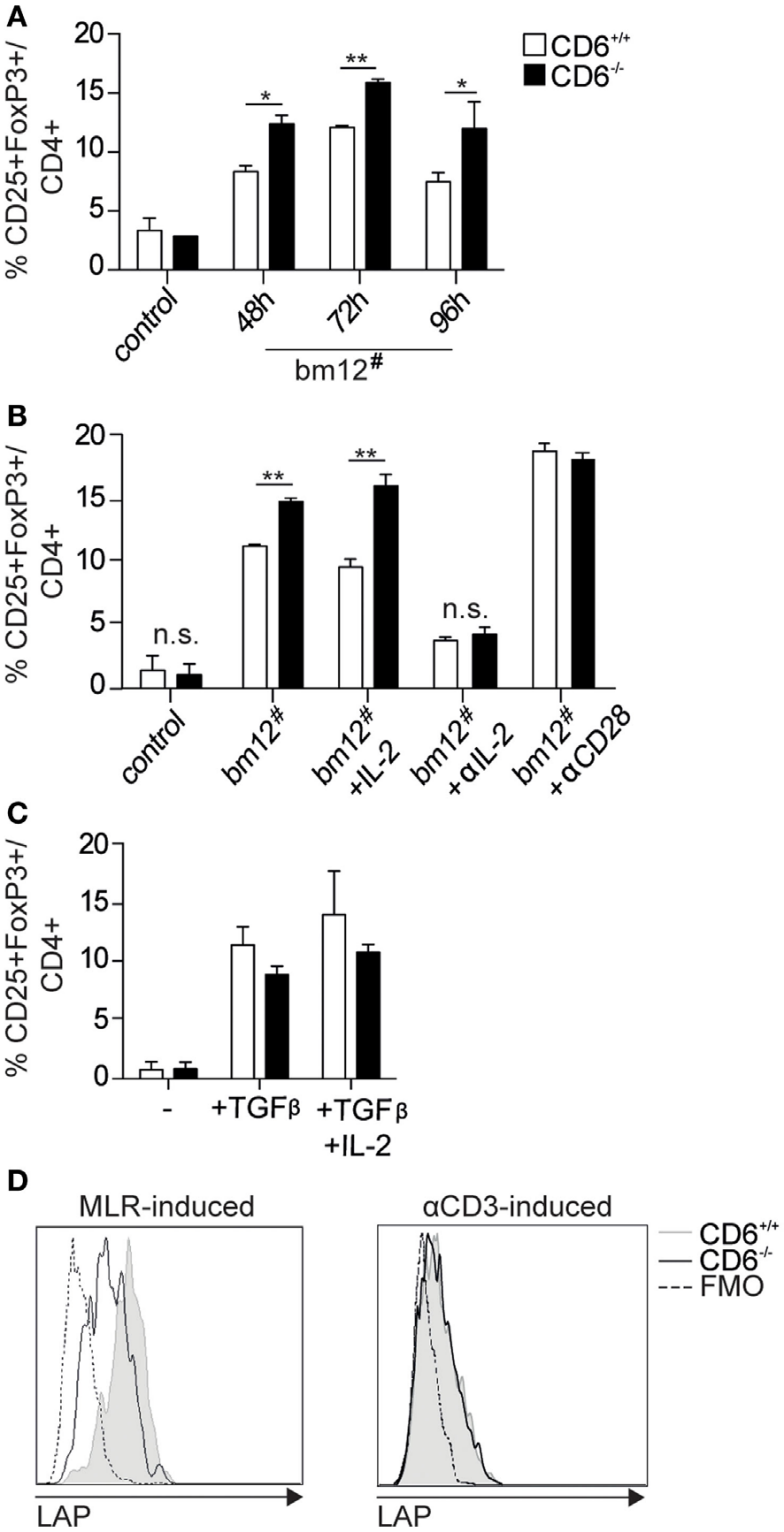
***In vitro* generation and functional analysis of spleen regulatory T cells from CD6^−/−^ mice**. **(A)** Purified CD4^+^ T cells (10^5^ cells/well) from CD6^−/−^ (solid bars) or CD6^+/+^ (empty bars) spleens were cocultured with irradiated bm12 splenocytes (bm12^#^) at a 2:1 ratio for different periods of time for further flow cytometry analysis. Data are shown as percentage (mean ± SD) of CD25^+^FoxP3^+^ cells on gated CD4^+^ cells from duplicates of one representative experiment of four performed. Control means the results of coculturing responder CD6^−/−^ or CD6^+/+^ cells with irradiated splenocytes from the same responder. **(B)** Same 72 h cocultures as in **(A)** in the absence (bm12^#^) or the presence of exogenously added IL-2 (+IL-2; 150 pg/mL), blocking anti-CD25 monoclonal antibody (mAb) (+αIL-2, 30 µg/mL) or agonistic anti-CD28 mAb (+ αCD28; 5 µg/mL). **(C)** Sorted (10^5^ cells/well) conventional CD4^+^CD25^−^ T cells (T_conv_) from CD6^−/−^ (solid bars) or CD6^+/+^ (empty bars) spleens were cultured for 3 days with plastic-bound anti-CD3 mAb (2 µg/mL) plus soluble anti-CD28 mAb (5 µg/mL) in the presence or absence of TGF-β (2 ng/mL) alone or plus IL-2 (20 ng/mL). **(D)** Flow cytometry analysis of latency-associated peptide (LAP) surface expression on CD4^+^ gated CD25^+^FoxP3^+^ T cells generated at 72 h as in **(A)** (*left*) or as in **(C)** (*right*). Shown is a representative experiment of four performed. n.s., not significant (Student’s *t-*test).

The MLR results on low proliferative T-cell responses in association with high IL-2 consumption led us to explore the *in vitro* induction of T_reg_ cells—a functionally relevant T cell subset known to consume a large fraction of the IL-2 produced by activated CD4^+^ T cells (35). To this end, the frequency of CD25^+^FoxP3^+^ cells was assessed by flow cytometry at different time points following coculture of purified CD4^+^ T-cells from CD6^−/−^ or CD6^+/+^ mice with allogeneic irradiated bm12 splenocytes. As illustrated by Figure 3A, statistically significant increased percentages of CD25^+^FoxP3^+^ cells were observed at all time points analyzed in MLR cultures using CD6^−/−^ cells as responders, compared to their CD6^+/+^ counterparts. These differences were maintained even in the presence of exogenously added IL-2 but were abrogated in the presence of blocking anti-CD25 mAb or agonist anti-CD28 mAb (Figure 3B). Interestingly, no statistically significant differences were not observed when assessed the frequency of CD25^+^FoxP3^+^ cells following stimulation of purified CD4^+^ T-cells under conventional T_reg_ induction conditions—that is, anti-CD3 plus anti-CD28 mAbs in the presence of T_reg_-inducing cytokines TGF-β and IL-2 (Figure 3C). This indicates both that (i) CD6 expression is relevant to proper T_reg_ induction during cell contact-dependent allogeneic stimulation and (ii) contact-independent and supraphysiological conventional conditions for *in vitro* induction of T_reg_ abrogates the differences found between CD6^−/−^ and CD6^+/+^ T cells.

**Figure 4:**
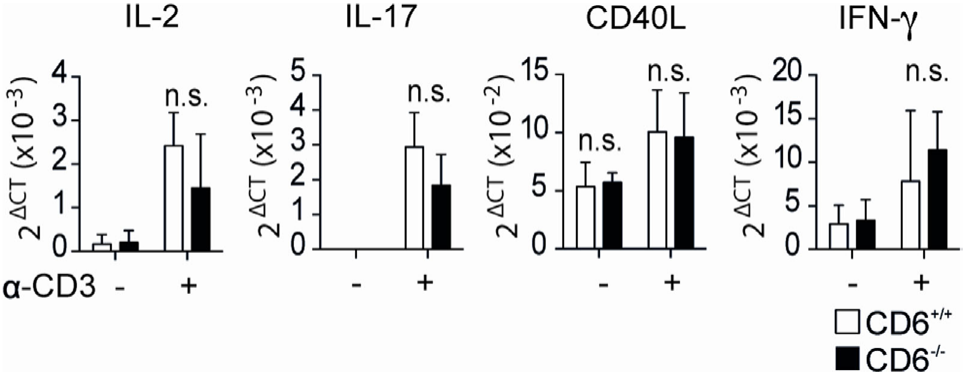
**Relative mRNA expression levels of *in vivo* isolated CD4^+^CD25^+^ cells from CD6^−/−^ and CD6^+/+^ mouse spleens following direct T-cell receptor complex cross-linking**. Sorted CD4^+^CD25^+^ T cells [regulatory T cells (T_reg_)] from CD6^−/−^ (*n* = 5, solid bars) and CD6^+/+^ (empty bars) were stimulated in anti-CD3 (1 µg/mL) coated plates. Relative mRNA expression for the indicated genes was measured after 7 h of stimulation by real-time PCR. n.s., not significant; *p < 0.05; **p < 0.01; ***p < 0.001; ****p < 0.0001 (Student’s *t*-test).

Moreover, surface expression of latency-associated peptide (LAP)—the N-terminal pro-peptide of the TGF-β precursor, which is considered to confer regulatory function to T cells and to be a selective marker of activated T_reg_ (36, 37)—was found to be lower on MLR-induced CD4^+^CD25^+^FoxP3^+^ T cells from CD6^−/−^ mice compared to those of CD6^+/+^ (Figure 3D, left). No differences regarding LAP expression were, however, observed on CD4^+^CD25^+^FoxP3^+^ T cells from CD6^−/−^ mice induced under the abovementioned conventional conditions (anti-CD3 + CD28 plus TGF-β/IL-2) (Figure 3D, right). These results are indicative of a relative defective activation and/or function of CD6^−/−^ T_reg_ cells under contact-dependent (MLR) but not contact-independent (anti-CD3 + CD28 plus TGF-β/IL-2) inductive conditions. Accordingly, direct TCR/CD3 cross-linking of CD4^+^CD25^+^ cells from CD6^−/−^ and CD6^+/+^ mice did not reveal statistically significant differences regarding relative mRNA expression levels of different lymphocyte activation-related genes (IL-2, IL-17, CD40L, and IFN-γ) (Figure 4).

### CD6^−/−^ Mice Show Exacerbated cGvHD-Induced Lupus-Like Disease

**Figure 5:**
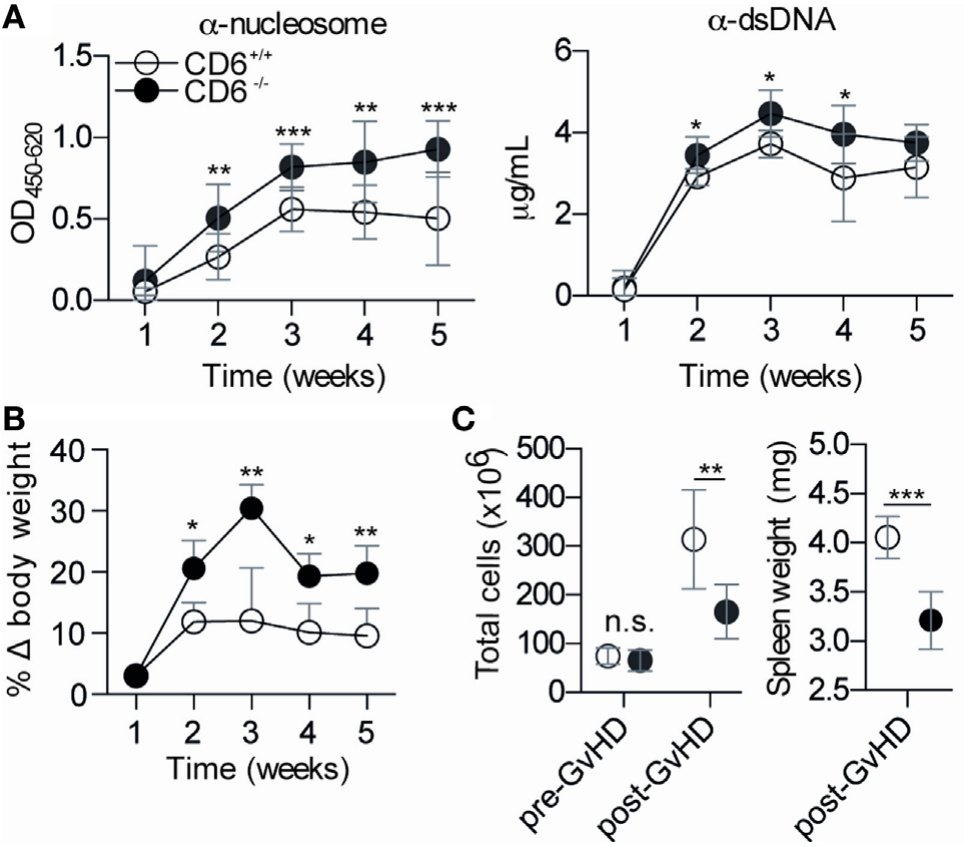
**Phenotypical characterization of CD6^−/−^ and CD6^+/+^ mice following chronic graft-versus-host disease (cGvHD)-induced lupus-like disease**. **(A)** ELISA determination of serum levels of autoantibodies against nucleosome (left) and dsDNA (right) at different weeks post-induction of cGvHD in CD6^−/−^ (*n* = 6; solid circles) and CD6^+/+^ (*n* = 6; empty circles) mice. Results are from one representative experiment of three performed and are expressed as mean ± SD of OD at 450 nm (anti-nucleosome) or concentration in microgram per milliliter (anti-dsDNA). **(B)** Percentage of weekly body weight variation (mean ± SD) with regard to initial weight from the same mice groups as in **(A)**. **(C)** *Left*, total spleen cell numbers pre and post (week 5) GvHD induction in the same mice groups as in (A). **(C)** *Right*, spleen weight (mg) at week 5 post cGvHD from same mice groups as in A. The results are the mean ± SD from one representative experiment of three performed. n.s., not significant; *p < 0.05; **p < 0.01; ***p < 0.001 (Student’s *t*-test).

**Figure 6:**
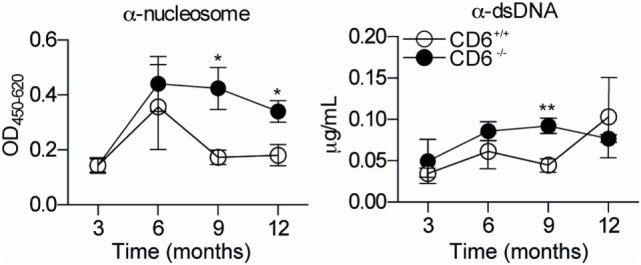
**Anti-dsDNA and anti-nucleosoma autoantibody titers in aged CD6^−/−^ and CD6^+/+^ mice**. Serum levels of autoantibodies against nucleosome (left-hand side) and dsDNA (right-hand side) from untreated CD6^−/−^ (*n* = 5, filled circles) and CD6^+/+^ (*n* = 5, empty circles) mice at 3, 6, 9, and 12 months of age were determined by ELISA. Data are expressed as mean ± SD of duplicates from a representative experiment of two performed. *p < 0.05; **p < 0.01 (Student’s *t*-test).

To further address the *in vivo* significance of the abovementioned MLR data (low lymphoproliferative responses and higher induction of putatively dysfunctional T_reg_ cells), a cGvHD-mediated autoimmune syndrome resembling systemic lupus erythematosus (30) was induced by challenging mice with coisogenic allogeneic cells. To this end, recipient CD6^−/−^ and CD6^+/+^ mice were injected (i.p.) with a single dose (10^8^ cells) of donor age/sex-matched allogeneic bm12 splenocytes (38). Weekly monitoring of serum autoantibody production revealed statistically significant increased levels of anti-dsDNA and anti-nucleosome in CD6^−/−^ mice compared to their CD6^+/+^ counterparts from the second week post-cell transfer (Figure 5A). This phenomenon was not only observed following cGvHD induction but also spontaneously with aging. As illustrated by Figure 6, low but still significant higher levels of autoantibodies were detected in aged CD6^−/−^ mice from their ninth month of life.

Moreover, body weight gain associated with serositis (e.g., ascites) and lymphoid organomegaly phenomena common in the cGvHD-induced model (30, 38) were also statistically increased in CD6^−/−^ mice (Figure 5B). The assessment of splenomegaly at the end of the follow-up period (week 5) showed, however, statistically significant lower total spleen cell numbers (Figure 5C, left) and spleen weight (Figure 5C, right) in CD6^−/−^ mice compared with their CD6^+/+^ counterparts. No differences regarding total spleen cell numbers (Figure 5C, left) were detected between CD6^+/+^ and CD6^−/−^ mice before induction of cGvHD-mediated lupus-like disease indicating that variances observed following bm12 cell injection could directly be related to differences in CD6 expression.

### CD6^−/−^ Mice Undergoing cGvHD-Induced Lupus-Like Disease Show Increased Frequency of Follicular B Cells and Dysfunctional T_reg_ Cells

**Figure 7:**
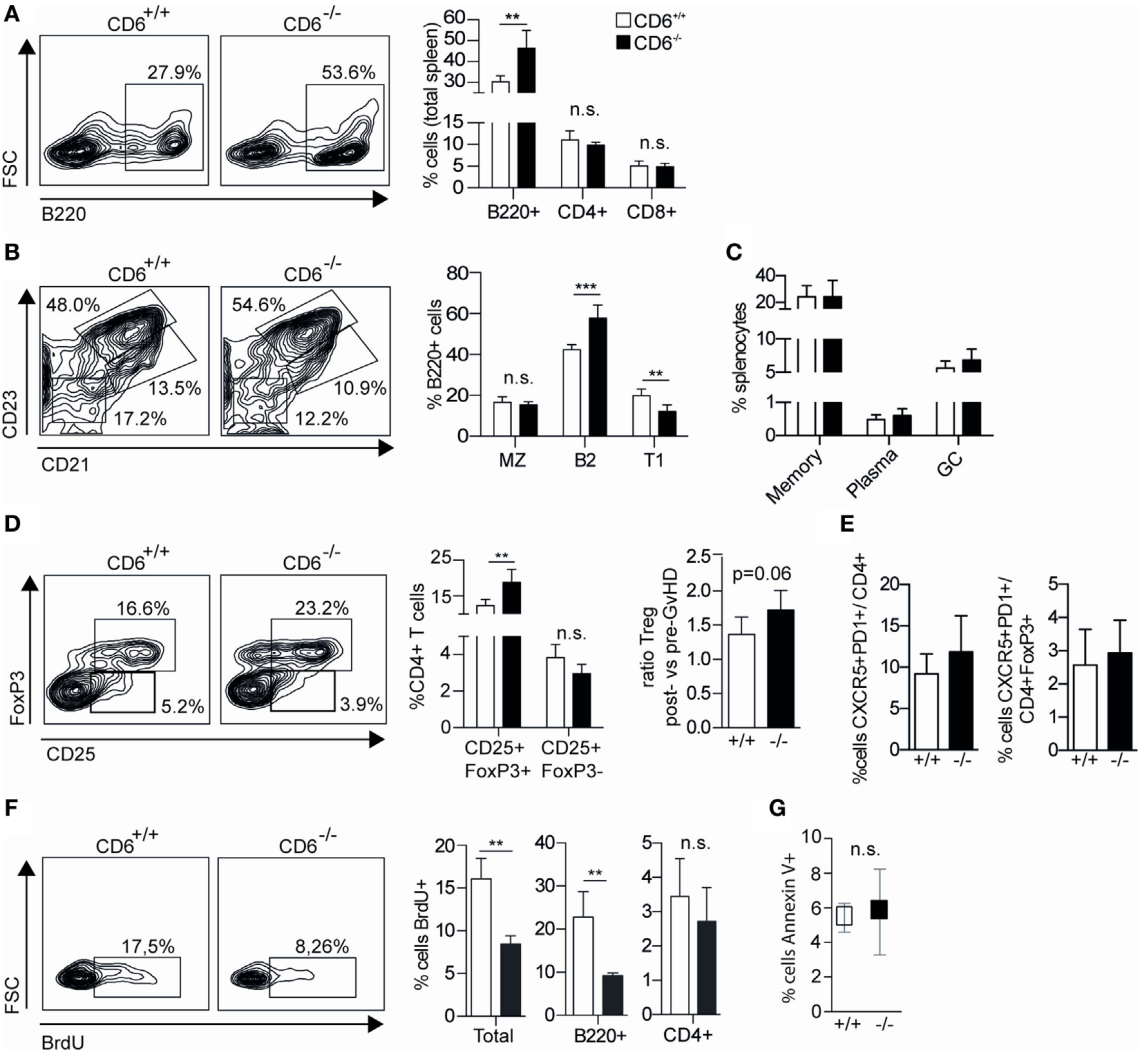
**Lymphocyte subset analysis of spleens from CD6^−/−^ and CD6^+/+^ mice undergoing chronic graft-versus-host disease (cGvHD)-induced lupus-like disease**. **(A)** Flow cytometry contour plots of B220^+^ cells and percentage (mean ± SD) of B220^+^, CD4^+^, and CD8^+^ cells from total spleens harvested at week 5 post cGvHD induction in CD6^−/−^ (*n* = **6**; solid bars) and CD6^+/+^ (*n* = **6**; empty bars) mice. **(B)** Flow cytometry contour plots and percentages (mean ± SD) of spleen B220^+^ cells with marginal zone B cells (MZ, CD21^+^CD23^low^), follicular (B2, CD21^+^CD23^+^), and transitional type 1 (T1, CD21^−^CD23^−^) phenotypes from the same groups of mice as in **(A)**. **(C)** Percentages (mean ± SD) of memory (CD19^+^CD138^−^IgD^−^IgM^−^), plasma (CD19^−^ CD138^+^), and germinal zone B-cells (CD19^+^GL-7^+^) from the same groups of mice as in **(A)**. **(D)** Percentages (mean ± SD) of follicular helper (T_FH_; CD4^−^CXCR5^+^PD1^+^) and follicular regulatory T cells (T_reg_) (T_FR_; CD4^−^CXCR5^+^PD1^+^ FoxP3^+^) from the same groups of mice as in **(A)**. **(E)** *Left*, Percentage (mean ± SD) of CD4^+^ spleen T cells with regulatory (CD25^+^FoxP3^+^) and activated (CD25^+^FoxP3^−^) phenotypes from the same groups of mice as in **(A)**. **(E)** *Right*, Increment in the percentage of spleen T_reg_ (CD4^+^CD25^+^FoxP3^+^) cells at week 5 postinduction of cGvHD in the same groups of mice as in **(A)**. Results are expressed as the ratio (mean ± SD) resulting from dividing the percentage of T_reg_ at week 5 by that at week 0. **(F)** Flow cytometry contour plots and percentage (mean ± SD) of BrdU^+^ cells from total, B220^+^ and CD4^+^ splenocytes at week 5 from the same groups of mice as in **(A)**. **(G)** Percentage (mean ± SD) of total spleen cells with late-stage apoptotic phenotype (Annexin V^+^ 7-AAD^+^) from the same groups of mice as in **(A)**. In all cases, data shown are from one representative experiment of three performed. n.s., not significant; BrdU, bromodesoxyuridine; **p < 0.01; ***p < 0.001 (Student’s *t*-test).

The flow cytometry analysis of major spleen lymphocyte subpopulations from week 5 post-GvHD induction revealed statistically significant increased percentages of B220^+^ (B) cells among CD6^−/−^ mice compared to CD6^+/+^ mice, while no significant differences were observed regarding the percentage of CD4^+^ and CD8^+^ T cells (Figure 7A). A more detailed analysis of B220^+^ spleen cell subsets revealed a significantly increased percentage of follicular cells (B2; CD21^+^CD23^hi^) concomitant with a significant reduction in that of transitional type 1 (T1; CD21^−^CD23^−^) B cells (Figure 7B). No significant differences were observed in the percentage of marginal zone B cells (MZ; CD21^+^CD23^low^), memory (CD138^−^IgD^−^IgM^−^), plasma (CD19^−^ CD138^+^) and germinal center (CD19^+^GL-7^+^) B cells (Figures 7B,C).

The subanalysis of spleen CD4^+^ T cells revealed a significantly increased percentage of cells with regulatory phenotype (CD25^+^FoxP3^+^) concomitant with a non-significant reduction of cells with activated/effector phenotype (CD25^+^FoxP3^−^) (Figure 7D). Although higher basal percentages of spleen CD4^+^CD25^+^FoxP3^+^ cells can be detected in CD6^−/−^ versus CD6^+/+^ mice pre-cGvHD induction (29), the increment at week 5 post cGvHD was higher for CD6^−/−^ mice compared to that of CD6^+/+^ ones (Figure 7D, right). No statistically significant differences were observed regarding follicular helper (T_FH_, CD4^−^CXCR5^+^PD1^+^) and follicular regulatory (T_FR_, CD4^−^CXCR5^+^PD1^+^FoxP3^+^) T-cell subsets (Figure 7E).

Further assessment of the underlying causes of lower splenomegaly found in CD6^−/−^ mice undergoing cGvHD was carried out by analyzing *in vivo* the levels of both proliferating and apoptotic spleen cells. With regard to the former, recipient CD6^+/+^ and CD6^−/−^ mice at week 5 post cGvHD induction were administered (i.p.) BrdU 15 h prior to sacrifice for further flow cytometry analysis. These *in vivo* BrdU-incorporation assays revealed a statistically significant lower percentage of BrdU^+^ spleen cells among CD6^−/−^ mice compared to CD6^+/+^ (Figure 7F). A more detailed analysis showed that both B220^+^ and CD4^+^ T cells from CD6^−/−^ mice incorporated less BrdU than those from CD6^+/+^ ones, though differences only reached statistical significance for B220^+^ cells. Investigation of spleen apoptotic cell levels by Annexin V and 7-AAD staining showed no statistically significant differences between CD6^−/−^ and CD6^+/+^ mice (Figure 7G), supporting lower proliferation as the main putative cause of reduced splenomegaly.

**Figure 8:**
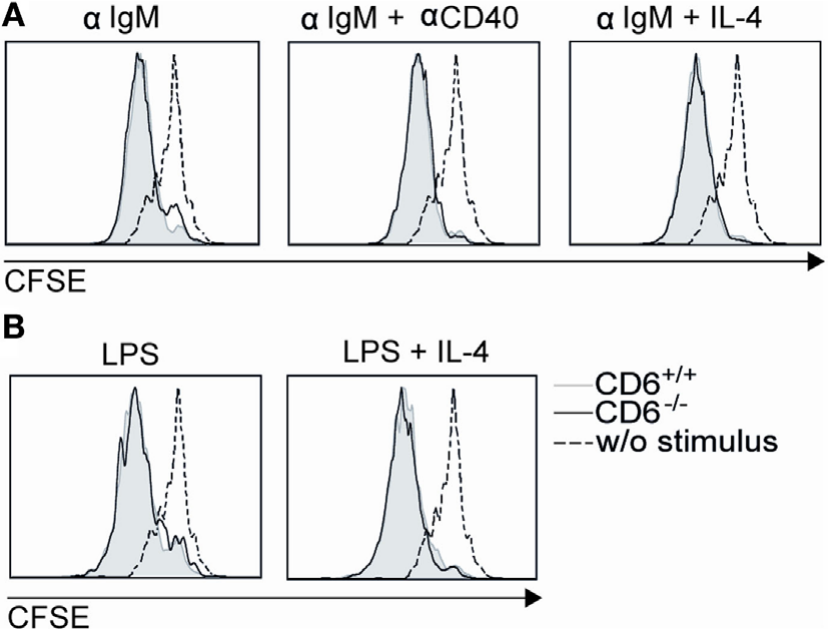
***In vitro* proliferative responses of B220^+^ cells from CD6^−/−^ and CD6^+/+^ mice to IgM cross-linking and lipopolysaccharide (LPS) stimulation**. **(A)** Negatively selected spleen B cells from CD6^−/−^ and CD6^+/+^ mice were CFSE-labeled and cultured for 3 days either unstimulated (w/o stimulus) or stimulated with F(ab′)_2_ anti-IgM (αIgM; 10 µg/mL) alone or in the presence of anti-CD40 (αCD40; 5 µg/mL) or IL-4 (10 ng/mL). **(B)** Same cells as in **(A)** were stimulated for 3 days with LPS (20 µg/mL) alone or plus IL-4 (30 ng/mL). Histograms show the CFSE fluorescence intensity of cells from a representative experiment of two performed.

Though spleen B cells are mostly CD6 negative, *in vitro* B-cell proliferation assays were also performed in order to exclude B-cell-intrinsic defects as responsible for low *in vivo* proliferation found in CD6^−/−^ mice following cGvHD-induced lupus-like. To this end, sorted B220^+^ splenocytes from CD6^−/−^ and CD6^+/+^ mice were CFSE-labeled and then stimulated with either F(ab’)_2_ anti-IgM or LPS in the presence or absence of anti-CD40 mAb or IL-4 costimulation. As shown in Figures 8A,B, similar B-cell proliferative responses were observed under all stimulatory conditions for CD6^−/−^ and CD6^+/+^ mice, thus arguing against the existence of B-cell-intrinsic defects associated with CD6 deficiency.

### Defective Suppressive Function of Spleen CD4^+^CD25^+^ T Cells from CD6^−/−^ Mice

**Figure 9:**
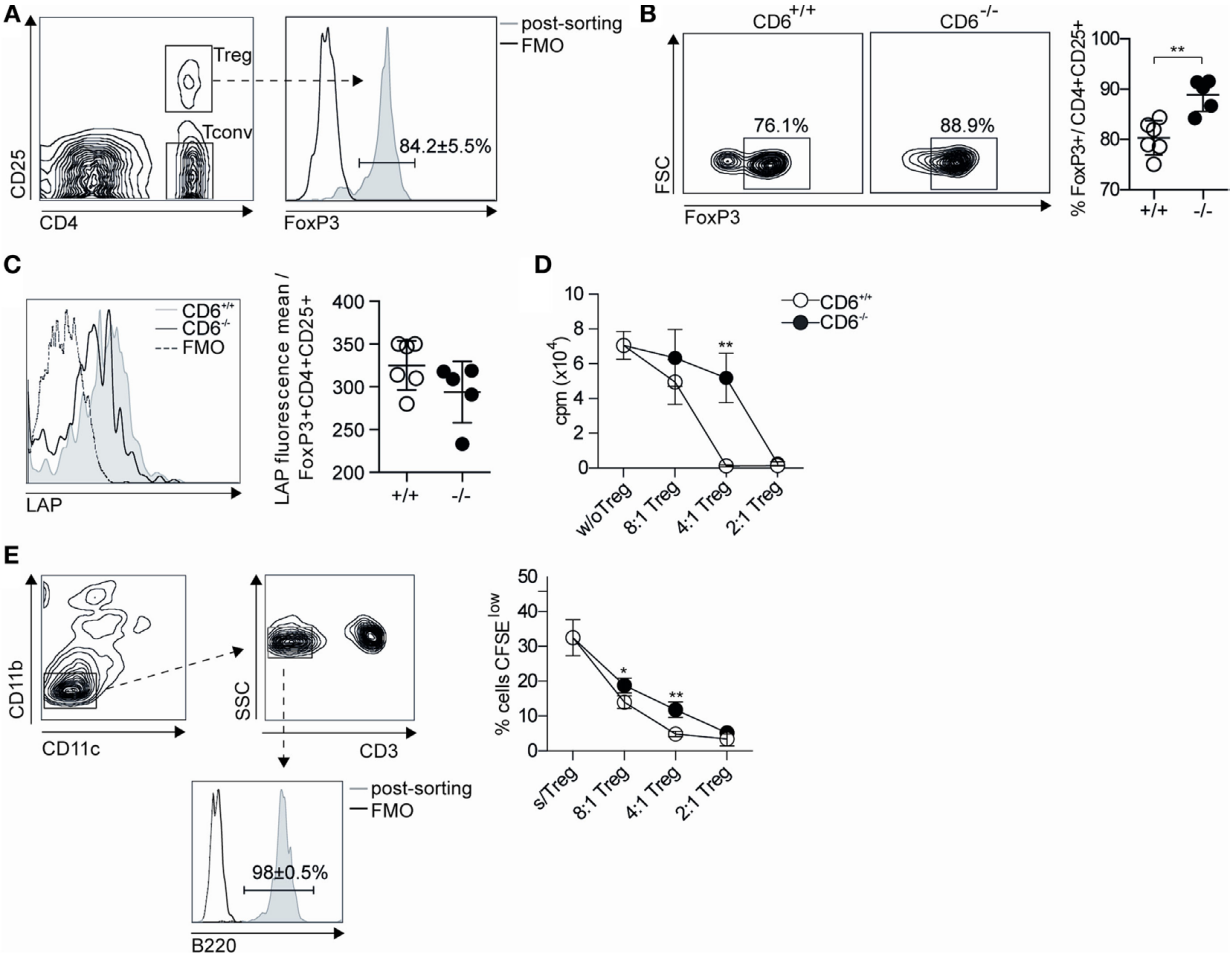
**Functional assessment of the suppressive activity of *in vivo* isolated CD4^+^ CD25^+^ spleen T cells from CD6^−/−^ and CD6^+/+^ mice**. **(A)** Density plot histogram showing the fluorescence intensity of conventional CD4^+^CD25^−^ (T_conv_) and regulatory CD4^+^CD25^+^ [regulatory T cells (T_reg_)] spleen T cells from CD6^+/+^ mice subjected to further FACS sorting. **(B)** Contour plot and percentages (mean ± SD) of FoxP3 expression in sorted CD4^+^CD25^+^ T cells 5 weeks after chronic graft-versus-host disease induction. **(C)** Flow cytometry analysis and percentages (mean ± SD) of latency-associated peptide (LAP) surface expression on CD4^+^ gated CD25^+^FoxP3^+^ T cells from spleens of untreated CD6^−/−^ and CD6^+/+^ mice. **(D)** Sorted T_conv_ cells from untreated CD6^+/+^ mice were cocultured at a 2:1 ratio with irradiated bm12 splenocytes alone or in the presence of sorted T_reg_ from CD6^−/−^ (solid circles) or CD6^+/+^ (empty circles) mice at week 5 post GvHD induction, added at the indicated T_conv_:T_reg_ ratios. Shown is the [^3^H]-thymidine incorporation (in cpm) after 5 days of coculture. **(E)** *Left*, density plot histogram showing the sorting strategy to isolate B220^+^ cells from CD6^+/+^ mice **(E)** *Right*. Sorted spleen B220^+^ cells from wild-type (CD6^+/+^) mice were CFSE-labeled and cultured for 3 days in the presence of F(ab’)_2_ anti-IgM fragments (10 µg/mL) alone or plus sorted CD4^+^CD25^hi^ (T_reg_) splenocytes from CD6^−/−^ (solid circles) or CD6^+/+^ (empty circles) mice, at the indicated B:T_reg_ ratios. The line chart show the percentage (mean ± SD) CFSE^low^ cells from a representative experiment of two performed. Data are mean ± SD of triplicate samples from one representative experiment of four performed. *p < 0.05; **p < 0.01 (Student’s *t*-test).

The fact that higher T_reg_ induction observed after *in vivo* (and *in vitro*) allogeneic stimulation did not prevent the presence of higher levels of circulating autoantibodies led to further assess the suppressive properties of CD6^−/−^ T_reg_ cells. To this end, sorted spleen CD4^+^CD25^+^ T cells (T_reg_) from either CD6^−/−^ or CD6^+/+^ mice at week 5 post cGvHD induction were subjected to flow cytometry analysis of intracellular and surface FoxP3 (Figure 9A) and LAP expression (Figure 9C), respectively. As shown in Figure 9B, sorted CD4^+^CD25^+^ T cells from CD6^−/−^ mice presented higher percentage of FoxP3 expression compared to CD6^+/+^ mice. Interestingly, lower surface expression levels of LAP were also observed for CD6^−/−^ mice (Figure 9C), which is suggestive of lower suppressive activity.

The same sorted CD4^+^CD25^+^ T cells were further tested in T_reg_ suppression assays. Accordingly, such cells were added at different cell ratios to cocultures of T_conv_ (CD4^+^CD25^−^) T cells from CD6^+/+^ mice used as responders, and irradiated total bm12 splenocytes used as stimulators. Proliferative responses measured by [^3^H]-thymidine incorporation at day 5 poststimulation showed a statistically significant lower suppressive activity for T_reg_ cells from CD6^−/−^ mice compared to their CD6^+/+^ counterparts (Figure 9D). Similar results were obtained when testing the suppressive activity of spleen T_reg_ from untreated (pre-cGvHD induction) CD6^−/−^ and CD6^+/+^ mice (data not shown), thus indicating that CD6 deficiency imposes a T_reg_ dysfunction which is not overcome post cGvHD induction.

The suppressive activity of T_reg_ cells from untreated (pre-cGvHD induction) CD6^−/−^ and CD6^+/+^ mice on B cells was also assessed by their coculture with CFSE-labeled sorted B220^+^ CD6^+/+^ splenocytes (Figure 9E, left) stimulated with F(ab′)_2_ anti-IgM. As shown in Figure 9E (right), significant lower suppressive activity was observed at different cell ratios for T_reg_ from CD6^−/−^ mice compared to their CD6^+/+^ counterparts. This indicates the existence of a general functional defect of T_reg_ in the absence of CD6 expression.

## Discussion

A balance between generation and function of effector/memory and T_reg_ is decisive for eliciting protective immune responses and keeping autoimmunity at bay (39). This report shows that CD6—a component of the TCR complex signaling machinery—influences such equilibrium by shaping the number and function of peripheral T_reg_.

The spontaneous generation of peripheral but not thymus-derived CD4^+^CD25^+^FoxP3^+^ cells in CD6^−/−^ mice has been described (29). Our *in vitro* (MLR assays) and *in vivo* (cGvHD-induced lupus-like model) results provide new clues as to why this happens. The experimental system described herein involves (i) direct cell-to-cell contact-mediated recognition of allogeneic MHC class II specificities and (ii) physiological-like stimulation conditions. CD6 is a complex molecule as its entire function rests on integrating CD6–CD166/ALCAM cell adhesion phenomena and the modulatory signals triggered by its cytoplasmic domain. Our MLR and cGvHD-induced lupus-like disease models allow for simultaneous evaluation of these two aspects of CD6 biology *in vitro* and *in vivo*. We observe low *in vitro* and *in vivo* T (and B) lymphoproliferative responses to allogeneic stimulation in CD6^−/−^ mice, which in turn exhibit exacerbated autoimmune events (namely, high titers of anti-dsDNA and anti-nucleosome autoantibodies characteristic of lupus-like disorders). These apparently opposing effects likely reflect the complex nature of CD6 function.

*In vitro* MLR assays reveal induction of (i) lower lymphoproliferative responses and (ii) higher generation of CD25^+^FoxP3^+^ cells with low LAP expression, in CD6^−/−^ T cells. These two findings are not reproducible via direct TCR/CD3 cross-linking and/or presence of costimulatory agents. The first one is in agreement with a critical role for CD6–CD166/ALCAM interaction in optimal IS formation and subsequent T-cell activation and proliferation (14–16). Unaffected proliferative responses of CD6^−/−^ T cells to direct TCR/CD3 cross-linking argue against cell-intrinsic T-cell defects and suggests suboptimal cell contact-dependent T–APC interactions in MLR-induced T-cell proliferation. The second finding indicates that low lympho-proliferation does not respond only to unsteady T–APC contacts but also to a simultaneous decrease in IL-2 availability associated with induced CD25^+^FoxP3^+^ cells with low activated (LAP) phenotype. Thus, absence of CD6-CD166/ALCAM interactions disrupts the equilibrium required in adaptive immune responses. This finding was masked by the use of standard stimulation conditions for *in vitro* T_reg_ generation (plate-bound anti-CD3 plus anti-CD28 mAbs and inducing cytokines), which further calls for the need of experimental systems that resemble, in as much as possible, real physiological settings (40).

The need for TCR/CD3-mediated signaling in homeostasis and T_reg_ suppressing action is well established (6). Also, low or interrupted TCR/CD3 stimulation favors FoxP3 expression both *in vivo* and *in vitro* (41, 42). Then, suboptimal T–APC contacts under CD6 deficiency may account for CD25^+^FoxP3^+^ cell induction. Moreover, ablation of the key TCR signal transducing effectors SLP-76 and PLC-γ1(43) are critical for T_reg_ function. Indeed, CD6 interacts directly with SLP-76 upon TCR engagement and phosphorylation by Zap70 (21, 25). Thus, altered TCR signaling due to inefficient recruitment of SLP-76 in CD6^−/−^ T cells may also be behind altered T_reg_ induction and/or function. Finally, the modulatory effects of CD6 signaling on TCR-mediated activation should be accounted for in this regard. Recent evidence points to a negative modulatory role for CD6, rather than a costimulatory one, in TCR signaling (20, 29). Even though CD6^−/−^ and CD6^+/+^ C57BL/6 mice during CD3 cross-linking-induced proliferative responses and mRNA expression, a CD6-mediated negative modulation of TCR signaling cannot be fully excluded. Indeed recent data from CD6^−/−^ DBA/1 mice show upregulated expression of early T-cell activation markers CD25 and CD69 together with reduced T-cell survival and proliferation following CD3 and CD28 co-cross-linking (44). Whether genetic differences between mouse strains are behind these apparent discrepancies would deserve further exploration.

The *in vivo* relevance of the MLR observations was confirmed in mice undergoing cGvHD-induced lupus-like disease. Splenomegaly at the end of the experimental period (week 5)—a hallmark of the cGvHD-induced autoimmunity model—was lower in CD6^−/−^ mice and explained by lower *in vivo* proliferation (low BrdU incorporation in the absence of vis-à-vis differences in apoptotic cell levels). Reduced *in vivo* proliferation was at the expense of the B-cell compartment and associated with higher frequency of follicular (B2) B cells. Importantly, *in vitro* proliferative responses of CD6^−/−^ B cells to direct cross-linking of the B-cell receptor complex were unaffected, excluding B cell-intrinsic defects. The reduced *in vivo* proliferation of CD6^−/−^ B cells may then respond to suboptimal cooperative T–B cell responses since (i) CD6 and CD166/ALCAM are constitutively expressed on T and B cells, respectively, and (ii) the CD6–CD166/ALCAM interaction is critical for optimal IS formation and stabilization.

Reduced B-cell proliferative responses do not prevent CD6^−/−^ mice from exacerbated autoantibody production after cGvHD induction (or spontaneously in aged animals) and correlate with the basal and inducible levels of T_reg_ cells with low LAP expression/suppressive function. Untreated CD6^−/−^mice show high levels of CD4^+^CD25^+^FoxP3^+^ T cells and low T- and B-cell proliferation suppression induced via CD3- (29) or IgM-cross-linking, together with increased autoantibody production in the absence of changes in stimulatory T_FH_ and inhibitory T_FR_ cell frequency. It is known that in response to T-dependent antigens a proportion of naïve T_reg_ express the follicular homing receptor CXCR5 (45) and that the resulting T_FR_ control the numbers and function of T_FH_, inhibiting non-antigen-specific B cells, including those carrying self-reactive receptors (45). Taken together, these observations suggest that dysfunctional T_reg_ cells migrating to B-cell areas may account for the hyperreactive autoantibody phenotype seen in CD6^−/−^ mice.

Following cGvHD-induced lupus-like disease, CD6^−/−^ mice showed higher induction of CD4^+^CD25^+^FoxP3^+^ cells with lower surface levels of the T_reg_ activation marker LAP, in line with their MLR-induced counterparts. Again, these cells from CD6^−/−^ mice were less efficient in suppressing the MLR-induced proliferation. No differences were observed in relative mRNA expression after direct TCR/CD3 cross-linking, which together with the absence of CD25^+^FoxP3^+^ cell and LAP surface expression during standard *in vitro* T_reg_ induction (anti-CD3 plus anti-CD28 in the presence of TGF-β and IL-2) argue against intrinsic defects in CD6^−/−^ T_reg_ cells. Thus, the reduced suppression observed in *ex vivo* isolated CD6^−/−^ cells with T_reg_ phenotype (both basally and post cGvHD induction) could account for defective cell contact-dependent events.

In summary, our results suggest first a direct involvement of CD6–CD166/ALCAM interaction in peripheral T-cell responses induced by cell contact-dependent antigen presentation; second, a role for CD6 in the maintenance of tolerance; and third, an incipient model for the molecular basis underlying CD6 targeting therapies, the latter in line with *CD6* susceptibility gene variants for multiple sclerosis (5, 6), Behçet’s disease (46), and CD6 deranged expression in Sjögren’s syndrome patients (47).

Recent approaches to CD6 targeting therapies include itolizumab, a humanized anti-CD6 mAb in psoriasic patients (2, 48), in view to extend its use to rheumatoid arthritis (49) and Sjögren’s syndrome (50). The latter is based on the *in vitro* inhibition by itolizumab of T-cell activation and differentiation to Th1 effector cells (51). However, mAb-induced CD6 cross-linking may also block CD6–CD166/ALCAM interaction by steric hindrance (52) or internalization (4), mimicking a “CD6-deficient like” phenotype. In this case, itolizumab would affect cell proliferative responses, in a similar way to what we observe in CD6^−/−^ mice.

Contrary to the situation in mice, human T_reg_ cells present low/negative CD6 expression (53), rendering itolizumab ineffective on this T-cell subset. Interestingly, the successful use of mAb-induced depletion of CD6^+^ T cells in prevention of GvHD in bone marrow transplants could be explained by the preservation of human T_reg_ cell subset in such a therapeutic setting (54). Moreover, the resulting CD6^−^ T cell population showed reduced alloreactivity in MLR assays (55) coincidental with our CD6^−/−^ data.

## Ethics Statement

Animals were housed in the accredited animal facility of the University of Barcelona, School of Medicine, and animal experimentations were performed after approval by and according to the guidelines of the University of Barcelona Animal Experimentation Ethical Committee for the use of laboratory animals, adhering to the Generalitat de Catalunya’s legislation on protection of such animals (protocol DAAM 7897 approved by CEEA-UB).

## Author Contributions

MC-F performed most of the experiments. FL, JL, and PE conceived the study and participated in the design and interpretation of results. MM-F, FA, JS, NA-B, TL, and NC participated in the experiments and drafting the manuscript. All the authors read and approved the final manuscript.

## Conflict of Interest Statement

The authors declare that the research was conducted in the absence of any commercial or financial relationships that could be construed as a potential conflict of interest.

## Abbreviations

APC, antigen-presenting cells; CD166/ALCAM, activated leukocyte cell adhesion molecule; B2, follicular B cells; bm12, B6.C-H-2^bm12^/KhEg mice; BrdU, bromodesoxiuridine; BSA, bovine seroalbumin; CD6^−/−^, CD6-deficient; cGvHD, chronic graft-versus-host disease; DC, dendritic cell; GC, germinal center B cells; i.p., intraperitoneal; IS, immunological synapse; LAP, latency-associated peptide; LPS, lipopolysaccharide; mAb, monoclonal antibody; MLR, mixed lymphocyte reaction; MZ, marginal zone B cells; PWM, Pokeweed mitogen; T1, transitional type 1 B cells; TCR, T-cell receptor; T_EM_, effector/memory T cells; T_reg_, regulatory T cells; T_conv_, conventional CD4^+^CD25^−^T cells; T_FH_, follicular helper T cell; T_FR_, follicular regulatory T cell.
